# Droplet based whole genome amplification for sequencing minute amounts of purified *Mycobacterium tuberculosis* DNA

**DOI:** 10.1038/s41598-024-60545-1

**Published:** 2024-04-30

**Authors:** Anzaan Dippenaar, Nabila Ismail, Tim H. Heupink, Melanie Grobbelaar, Johannes Loubser, Annelies Van Rie, Robin M. Warren

**Affiliations:** 1 Tuberculosis Omics Research Consortium, Department of Family Medicine and Population Health, Global Health Institute, Faculty of Medicine and Health Sciences, University of Antwerp, Antwerp, Belgium; 2 South African Medical Research Council Centre for Tuberculosis Research, Division of Molecular Biology and Human Genetics, Faculty of Medicine and Health Sciences, Stellenbosch University, Cape Town, South Africa

**Keywords:** Tuberculosis, Whole genome amplification, WGS, Sequencing, Droplet generator, Clinical microbiology, Genetics, Molecular biology, Medical research, Molecular medicine

## Abstract

Implementation of whole genome sequencing (WGS) for patient care is hindered by limited *Mycobacterium tuberculosis* (*Mtb*) in clinical specimens and slow *Mtb* growth. We evaluated droplet multiple displacement amplification (dMDA) for amplification of minute amounts of *Mtb* DNA to enable WGS as an alternative to other *Mtb* enrichment methods. Purified genomic *Mtb*-DNA (0.1, 0.5, 1, and 5 pg) was encapsulated and amplified using the Samplix Xdrop-instrument and sequenced alongside a control sample using standard Illumina protocols followed by MAGMA-analysis. The control and 5 pg input dMDA samples underwent nanopore sequencing followed by Nanoseq and TB-profiler analysis. dMDA generated 105-2400 ng DNA from the 0.1-5 pg input DNA, respectively. Followed by Illumina WGS, dMDA raised mean sequencing depth from 7 × for 0.1 pg input DNA to ≥ 60 × for 5 pg input and the control sample. Bioinformatic analysis revealed a high number of false positive and false negative variants when amplifying ≤ 0.5 pg input DNA. Nanopore sequencing of the 5 pg dMDA sample presented excellent coverage depth, breadth, and accurate strain characterization, albeit elevated false positive and false negative variants compared to Illumina-sequenced dMDA sample with identical *Mtb* DNA input. dMDA coupled with Illumina WGS for samples with ≥ 5 pg purified *Mtb* DNA, equating to approximately 1000 copies of the *Mtb* genome, offers precision for drug resistance, phylogeny, and transmission insights.

## Introduction

Tuberculosis (TB) remains a major global health concern, as it continues to cause a high burden of disease with an estimated 10 million new cases, including 450,000 rifampicin-resistant cases, and 1.6 million deaths in 2021^1^. Despite being a curable disease, effective diagnosis remains a challenge, hindering prompt initiation of appropriate treatment. The current gold standard for TB diagnosis involves microbiological detection by a rapid molecular test followed by phenotypic drug susceptibility testing (pDST) of the *Mycobacterium tuberculosis* (*Mtb*) isolate to select the most effective treatment^2^. This process is time-consuming and can delay crucial treatment decisions.

Targeted next-generation sequencing (tNGS) and whole-genome sequencing (WGS) have emerged as an alternative approach to pDST, providing information on multiple drug resistance markers, including those conferring resistance to new and repurposed drugs^2,3^. Nonetheless, the implementation of sequencing technologies into routine clinical practice remains limited^4^. WGS typically requires 100–1000 ng of high-quality DNA^5,6^, necessitating a culture step or costly DNA enrichment methods, which are impractical for routine laboratory settings^7,8^. Furthermore, the enrichment process can result in the selection of subpopulations, altering the population structure from the original sample and potentially affecting the accurate detection of the resistance profile^4,8^. Additionally, viable non-culturable persister organisms, hypothesized to cause relapse after insufficient treatment, may be missed by WGS of (sub)-cultured isolates^4,9^. While tNGS can be performed without culture or an *Mtb* enriching step, the quantity of DNA extracted from clinical specimens can be insufficient for tNGS, especially for paucibacillary samples such as smear-negative sputum samples or extrapulmonary samples^10,11^.

Whole genome amplification (WGA) has emerged as a promising technique that could overcome these challenges. Among the various WGA methods, Multiple Displacement Amplification (MDA) stands out as a robust procedure that amplifies all DNA present in a sample and generates large-sized products with a low error frequency^12^. Compared to conventional PCR amplification, MDA does not employ sequence-specific primers but utilizes random hexamer primers that anneal to the DNA template, and DNA synthesis is carried out by a high-fidelity enzyme, typically phi29 DNA polymerase. The displaced DNA strands act as templates for additional primer extensions, with multiple iterations of strand displacement and primer extension resulting in networks of branched DNA structures. Studies have indicated that MDA has the capacity to amplify genomic DNA uniformly by over a billionfold, achieving comprehensive genome coverage without substantial bias concerning the initial sequence proportions^13,14^.

MDA has found widespread application in amplifying genomic DNA from diverse organisms. Its application to *Mtb* has been limited as the currently used lysis methods struggle to efficiently break down the cell wall, leading to suboptimal DNA yields extracted from paucibacillary samples which subsequently affects the suitability of the extracted DNA for the MDA reaction^15^. Additionally, the GC-rich nature of the *Mtb* genome can pose challenges if the DNA polymerase lacks high fidelity, and the ability to amplify all DNA present in a clinical sample raises concerns about potential contamination and metagenomic amplification when processing samples without an effective *Mtb* enrichment or contaminant depletion step^16^.

To address these limitations and explore the feasibility of WGS from samples with minute amounts of initial input *Mtb* DNA, we evaluated droplet MDA (dMDA), which combines MDA with droplet partitioning for unbiased WGA. We employed the novel Xdrop technology, a microfluidic-based system (Samplix, Birkerød, Denmark) that disperses in-solution DNA into single-emulsion microdroplets and amplifies individual DNA molecules without competition between amplification reactions, thus minimizing the risk of intermolecular chimeric molecules^17^. We hypothesized that the Xdrop dMDA method could accurately and efficiently amplify *Mtb* DNA and enable WGS from small amounts of DNA equivalent to what will be found in paucibacillary samples. In this manuscript, we present proof-of-concept pilot data on the application of dMDA to picogram-amounts of *Mtb* DNA and discuss its potential for improving TB diagnosis and drug resistance detection.

## Materials and methods

### Sample selection and DNA dilution

**Figure 1:**
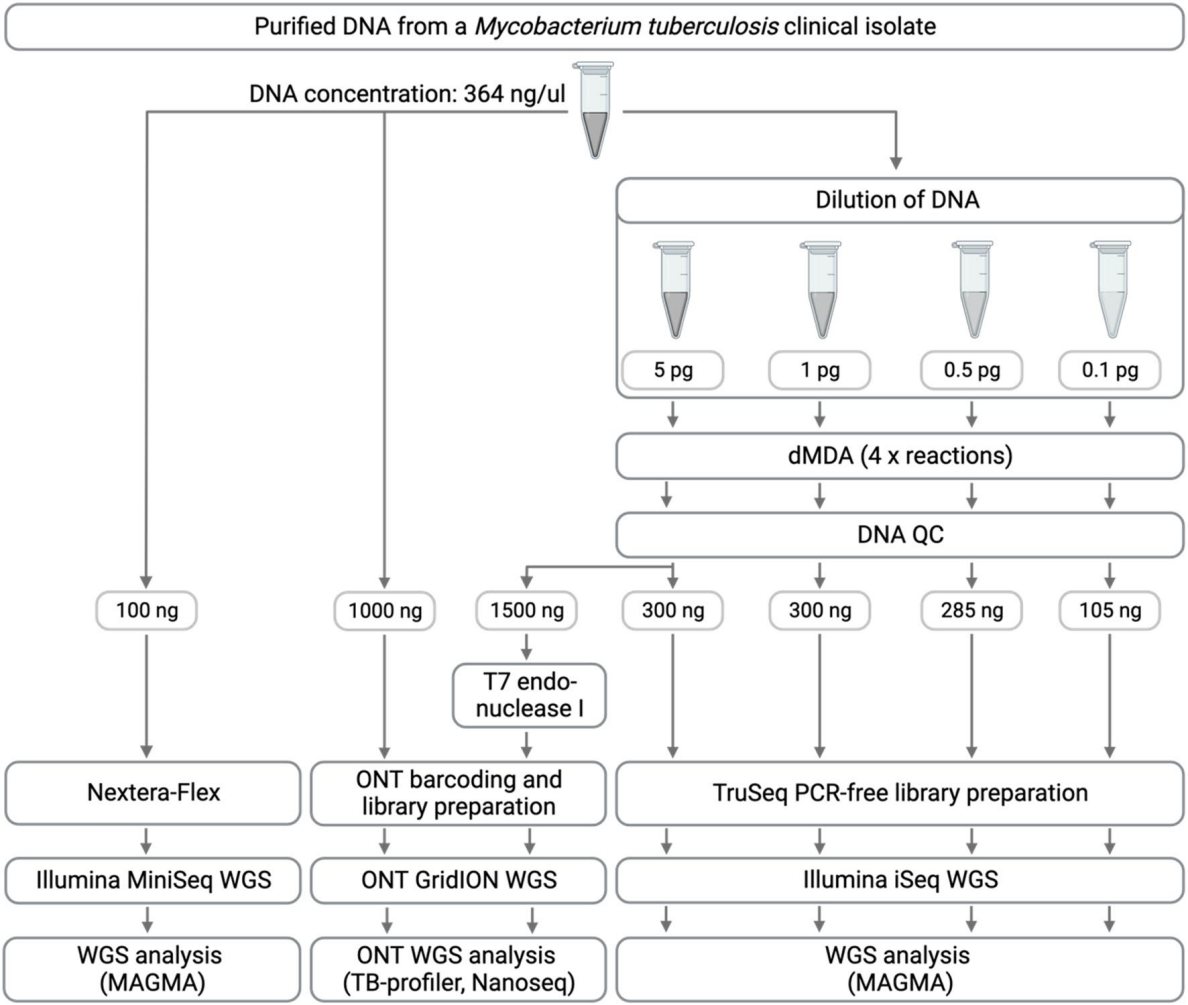
Overview of study design and experimental procedures. High-quality purified DNA (364 ng/μl) extracted from a Lineage 2 *Mtb* clinical isolate served as a positive control (Illumina MiniSeq WGS). The DNA was diluted to be used as starting material for four dMDA reactions (5 pg, 1 pg, 0.5 pg, and 0.1 pg, respectively). The DNA after dMDA was quantified using a Quantus fluorometer, assessed for fragmentation using the TapeStation™, and subsequently used for library preparation and Illumina WGS on an iSeq instrument. Data analysis was done using the MAGMA pipeline. ONT WGS was done on the control and 5 pg DNA input dMDA sample and the ONT data analysis was done using TB-profiler. *Figure created with* *BioRender.com. Abbreviations: dMDA* = *droplet multiple displacement amplification; WGA* = *whole genome amplification; WGS* = *whole genome sequencing.*

For this study, we selected a pan-susceptible clinical *Mtb* isolate belonging to Lineage 2.2.1.1. The DNA was extracted using the cetyltrimethylammonium bromide (CTAB) method^18^, quantified using a Quantus fluorometer (Promega), and DNA fragment size distribution was determined with the TapeStation™ System (Agilent Technologies Inc.) using Genomic DNA ScreenTape. Input amounts of 0.1 pg, 0.5 pg, 1 pg, and 5 pg of *Mtb* DNA were prepared for dMDA, each in 2 μl in nuclease-free water (Fig. 1). These input quantities correspond to approximately 20 to1000 copies of *Mtb* genomes (Table 1), considering the estimated size of the *Mtb* genome to be approximately 4.4 Mb (https://cels.uri.edu/gsc/cndna.html).

**Table 1:** DNA yield and Illumina whole genome sequence statistics for samples with four different input *Mycobacterium tuberculosis* DNA amounts processed by droplet multiple displacement amplification.

	Sample type					
	Positive control (no dMDA)	Negative control	dMDA samples with increasing *Mtb* DNA input material (pg)			
	–	0	0.1	0.5	1	5
**dMDA DNA product characterisation**						
dMDA DNA total yield (ng) in 15 ul reaction	–	NA	105	285	1140	2400
dMDA DNA concentration (ng/μl)	–	0.1	7	19	76	160
TapeStation™ DNA Integrity number	6.2	–	8.9	7.5	7.8	7.6
**WGS output statistics**						
Number of read pairs	1 240 462	–	302 099	1 045 165	1 052 951	1 416 070
Average base quality	35.6	–	34.4	34.3	34.3	34.3
Mean genome-wide depth of coverage (×)	65	–	7	33	43	61
Genome with ≥ 5× depth of coverage (%)	98	–	44	92	97	98
Reads mapping to H37Rv reference genome (%)	89	–	52	64	76	78
**WGS results**						
Lineage	L2.2.1.1	–	L2.2.1.1	L2.2.1.1	L2.2.1.1	L2.2.1.1
Drug resistance profile	Pan-susceptible	–	Pan-susceptible	Pan-susceptible	Pan-susceptible	Pan-susceptible

*dMDA* *droplet multiple displacement amplification, Mtb* *Mycobacterium tuberculosis, DIN* *DNA integrity number, WGS* *whole genome sequencing, NA* *not applicable.*

### Droplet multiple displacement amplification

Using the dMDA kit (Samplix, Denmark), separate dMDA reactions were prepared for each starting amount of *Mtb* DNA (0.1 – 5 pg), alongside a negative control containing nuclease-free water without DNA template. Each reaction consisted of 4 μl of 5 × dMDA mix, 13 μl nuclease-free molecular grade water, and 2 μl of diluted DNA. The samples were denatured at 95 °C for 2 min, followed by cooling, after which 1 μl of dMDA enzyme (phi29 DNA polymerase, 0.125 U/μl) was added to each reaction. The reaction mixtures were loaded into dMDA cartridges (Samplix, Denmark) and partitioned in approximately 65,000 single emulsion droplets by Xdrop™, reaching an approximate encapsulation efficiency of ± 95%. Single emulsion droplets were incubated for 16 h at 30 °C, after which the reactions were stopped by incubating at 65 °C for 10 min and stored at 4 °C. The amplified DNA, recovered in 15 μl for each dMDA reaction, was isolated by disrupting the droplets using 20 μl of "break" solution and 1 μl of break colour. The amplified DNA recovered from each of the four samples and the negative control was quantified using Quantus, and the DNA fragment size distribution was assessed using the TapeStation™.

### Whole genome sequencing

No library was prepared for the negative control dMDA sample given that there were negligible amounts of DNA found by Quantus. WGS for the sample not processed by dMDA (positive control) was generated using the Nextera Flex kit and an Illumina MiniSeq instrument. The dMDA products were processed using the Illumina TrueSeq PCR-free Library Preparation Protocol, SEQ100 v2 cartridges and Illimuna iSeq100 desktop instrument. The entire reaction volume of the dMDA product was used for the samples with 0.1 pg and 0.5 pg input DNA due to the low amplification yield. For the samples with 1 pg and 5 pg inputs, only 300 ng of the dMDA product was used. The remaining material of the 5 pg input dMDA sample and the positive control was used to generate long-read sequencing data using the Oxford Nanopore Technologies (ONT) sequencing platform (Oxford Nanopore Inc.). For library preparation and multiplexing, the ligation sequencing kit (SQK-LSK109) with the Native Barcoding Expansion kit (EXP-NBD104) was used according to the manufacturer’s instructions. For the the dMDA sample, 1.5 μg was treated using T7 Endonuclease I followed by size selection, end-repair, barcoding, and adaptor ligation. The 1000 ng genomic DNA not processed by dMDA was not treated with T7 endonuclease I. After library generation, the pooled sequence library was loaded onto a GridION flow cell 9.4.1 (5–50 fmol) and run for 16 h under standard conditions.

### Data analysis

The raw Illumina WGS reads (FASTQ) from the four dMDA and positive control sample were analysed using the MAGMA bioinformatics pipeline which aligns reads to the *Mtb* H37Rv (NC000962.3) reference genome for variant identification^19,20^. Variants called through the major variants workflow of MAGMA were used for subsequent analyses. However, allele frequencies of the raw unfiltered variants were plotted ( Appendix 1, Figure S1). The BAM files were analysed using Qualimap2 to obtain basic WGS-mapping statistics as part of the MAGMA pipeline^21^. For each of the four samples, single nucleotide polymorphisms (SNPs) and insertions and deletions (indels) identified were compared to those present in the positive control sample to identify false negative variants and false positive variants that may have been introduced by dMDA or sequencing. Pairwise comparisons were performed twice, once including and once excluding difficult-to-map repeat regions, referred to as ‘complex regions’^19,22^. The complex regions list employed by MAGMA correlates with that defined and incorporated into the Unified Analysis Variant Pipeline (UVP)^22^. The assessment of false positive and false negative variants included an investigation into their proportion within *pe/ppe* genes relative to other genomic regions categorised as complex regions (Appendix 1, Table S1). In addition, Kraken 2 was used to classify sequencing reads, including raw FASTQ files and unmapped reads extracted from the BAM files (Appendix 1, Table S2)^23^.

The raw ONT data (FAST5) was subjected to real-time base calling using MinKNOW software (v21.05.12) on super high accuracy, filtered on Phred Quality score 10, and saved in the FASTQ format. Barcodes and adaptors were removed in this process. The ONT data were analysed using TB-profiler for identification of drug-resistance conferring variants and lineage identification, specifying ‘nanopore’ as the platform^24,25^. Additionally, the ONT data were analysed with nfcore/nanoseq (v3.1.0)^26,27^, specifying the *Mtb* H37Rv (NC_000962.3) sequence as reference, and the resulting variants, identified with Deepvariant (v1.4.0)^28^, were used for pairwise comparison and the mapping files were used to estimate the mapping statistics using Qualimap 2^21,29^.

## Results

### DNA yield from dMDA

The DNA yield, as measured by Quantus, generated by the dMDA process in the recovered 15 μl reaction volume was negligible (0.1 ng/μl) for the negative control (molecular-grade nuclease-free water) and increased from 105 ng for 0.1 pg input DNA to 2400 ng for 5 pg input DNA (Table 1). This corresponds to a concentration of the dMDA product ranging from 7 ng/μl for 0.1 ng input DNA to 160 ng/μl for 5 pg input DNA. The DNA integrity number (DIN), assessed through TapeStation™ analysis, varied from 8.9 for the 0.1 pg input sample to 7.6 for the 5 pg input sample after dMDA, with the positive control sample exhibiting a DIN of 6.2. The DNA concentration readout of 0.1 ng/μl for the negative control likely stems from the random hexamer primers and dNTPs in the dMDA reagents.

### WGS statistics (Illumina)

WGS of the dMDA DNA products resulted in increasing numbers of Illumina iSeq read pairs as the initial dMDA *Mtb* DNA input amount increased: from 302,099 read pairs for 0.1 pg to 1,416,070 read pairs for 5 pg input DNA (Table 1). The average base quality score for all dMDA-WGS samples was 34.3, only slightly lower than that of the positive control sample (35.2). The mean sequencing depth increased from 7 × for the 0.1 pg input DNA to 61 × for the 5 pg input DNA sample, similar to the 65 × depth obtained for the positive control sample. Overall, similar trends in the depth of coverage distribution were observed, with a prominent (± 350 kb) duplication of the dosR-region evident in the coverage plots of all samples, indicating its preservation in the dMDA samples (Appendix 1, Figure S2). The average breadth of coverage at ≥ 5 × was low (44%) when only 0.1 pg input DNA was used, increased to 92% when 0.5 pg was used, and was similar (≥ 97%) to the positive control when 1 or 5 pg input DNA was used. Similarly, the percentage of reads that mapped to the *Mtb* H37Rv reference genome increased with increasing sample input DNA, from 52% for 0.1 pg input DNA to 78% for 5 pg input DNA, but this remained below the 89% reads that could be mapped to the reference genome for the positive control sample. Upon analysis of the raw FASTQ reads for each sample and the control using the taxonomic classification tool Kraken 2, we observed an increasing number of unclassified reads, indicating that the tool could not assign the taxonomic origin of an increased proportion of the reads, as the input *Mtb* DNA amount decreased (Appendix 1, Table S2). This observation also correlates with a decrease in the percentage of mapped reads as the input *Mtb*-DNA amount decreases. Independent of the input amount, the MAGMA pipeline accurately identified the lineage of the strain as Lineage 2.2.1.1 and correctly identified the strain as pan-susceptible.

### WGS accuracy: false positive and false negative variants introduced by MDA (Illumina)

In comparison to the *Mtb* H37Rv reference genome, a total of 1290 variants were identified in the positive control sample when complex regions were excluded from the analysis: 1180 SNPs and 110 indels. When all variants identified in the WGS analysis of the positive control sample were considered true variants, then one (0.08%) SNP was a false negative SNP when 5 pg input DNA was used for dMDA, meaning that only one SNP present in the positive control was not detected by the WGS analysis of the dMDA sample. The number of false negative variants increased with decreasing input DNA: 9 (0.70%) (6 SNPs and 3 indels) in the 1 pg input sample, 41 (3.01%) (34 SNPs and 7 indels) in the 0.5 pg input sample and 548 (59.79%) (501 SNPs and 47 indels) in the 0.1 pg input sample (Table 2). Similarly, the number of false positive variants, i.e. variants were detected by WGS in the dMDA samples but not in the positive control sample, increased with decreasing DNA input for the dMDA process: 4 (0.31%) (1 SNP and 3 indels) in the 5 pg input DNA sample, 13 (1.00%) (11 SNPs and 2 indels) in the 1 pg input DNA sample, 114 (8.36%) (106 SNPs and 8 indels) in the 0.5 pg input sample and 175 (19.08%) (183 SNPs and 12 indels) in the 0.1 pg input sample.

**Table 2:** Number of false positive and false negative variants (SNPs and indels) identified in WGA-WGS samples when using the MAGMA pipeline for analysis of Illumina sequencing reads.

		Positive control	dMDA processed samples			
Amount of *Mtb* DNA used as input for the dMDA process (pg)			0.1	0.5	1	5
**Excluding complex regions**	**Total number of variants identified**	**1290**	**917**	**1363**	**1294**	**1293**
	Number of SNPs	1180	842	1252	1185	1180
	Number of indels	110	75	111	109	113
	Number of false negative* variants (%)		**548 (59.76%)**	**41 (3.01%)**	**9 (0.70%)**	**1 (0.08%)**
	False negative SNPs		501 (54.63%)	34 (2.49%)	6 (0.45%)	1 (0.08%)
	False negative indels		47 (5.13%)	7 (0.51%)	3 (0.23%)	0 (0%)
	Number of false positive* variants (%)		**175 (19.08%)**	**114 (8.36%)**	**13 (1.00%)**	**4 (0.31%)**
	False positive SNPs		163 (17.78%)	106 (7.78%)	11 (0.85%)	1 (0.08%)
	False positive indels		12 (1.31%)	8 (0.59%)	2 (0.15%)	3 (0.23%)
**Including complex regions**	**Total number variants identified**	**1655**	**1106**	**1688**	**1646**	**1657**
	Number of SNPs	1503	1014	1544	1502	1503
	Number of indels	152	92	144	144	154
	Number of false negative* variants (%)		**758 (68.54%)**	**113 (6.69%)**	**54 (3.28%)**	**31(1.87%)**
	False negative SNPs		680 (61.48%)	90 (5.33%)	39 (2.37%)	23 (1.39%)
	False negative indels		78 (7.05%)	23 (1.36%)	15 (0.91%)	8 (0.48%)
	Number of false positive* variants (%)		**209 (18.90%)**	**146 (8.65%)**	**45 (2.73%)**	**33 (1.99%)**
	False positive SNPs		191 (17.27%)	131 (7.76%)	38 (2.31%)	23 (1.39%)
	False positive indels		18 (1.63%)	15 (0.89%)	7 (0.43%)	10 (0.60%)

*In this analysis, all variants identified by WGS analysis of the positive control sample were considered to be true variants, false positive variants were defined as variants observed in the dMDA-WGS sample but not in the positive control; false negative variants were defined as variants observed in the positive control sample but not detected in the dMDA-WGS sample.

The inclusion of complex genomic regions in the analysis increased the total number of variants detected by WGS in the positive control sample from 1290 (1180 SNPs and 110 indels) to 1655 (1503 SNPs and 152 indels). The number of false negative variants increased as the amount of input DNA for the dMDA reactions decreased: from 31 (1.87%) variants (23 SNPs and 8 indels) in the 5 pg input DNA sample, to 54 (3.28%) (39 SNPs and 15 indels) in the 1 pg input sample, 113 (6.69%) (90 SNPs and 23 indels) in the 0.5 pg input sample and 758 (68.54%) (680 SNPs and 78 indels) in the 0.1 pg input sample(Table 2). The number of false positive variants also increased with decreasing DNA input for the dMDA process, from 33 (1.99%) (23 SNPs and 10 indels) in the 5 pg input DNA sample to 45 (2.73%) (38 SNPs and 7 indels) in the 1 pg input DNA sample, 146 (8.65%) (131 SNPs and 15 indels) in the 0.5 pg input sample and 209 (18.90%) (191 SNPs and 18 indels) in the 0.1 pg input sample. For most of the samples, in both the analysis including and excluding complex genomic regions, the proportion of errors was larger when it came to indels compared to SNPs.

### Comparison of ONT and Illumina sequencing

The data generated by ONT sequencing exhibited high depth, reaching 147 × for the positive control and 452 × for the 5 pg input sample. Additionally, the breadth of coverage was extensive, with both the positive control and diluted sample achieving at least 99.28% and 99.35% coverage at 5 × or greater (Table 3). For both samples, more than 99% of the reads generated were mapped to the *Mtb* H37Rv reference genome. TB-profiler accurately identified the lineage of the strain as Lineage 2.2.1.1 in both samples and confirmed the pan-susceptible nature of the strain. During the whole-genome variant calling process, which included complex genomic regions, analysis of the 5 pg dMDA sample using Nanoseq revealed 65 (4.07%) false positive variants, encompassing 50 SNPs and 15 indels, along with 53 (3.32%) false negative variants, consisting of 32 SNPs and 21 indels. Subsequently, when the whole-genome variant calling was performed while excluding complex genomic regions in the same 5 pg dMDA sample with Nanoseq, 23 (1.86%) false positive variants (comprising 15 SNPs and 8 indels) and 24 (1.94%) false negative variants (comprising 8 SNPs and 16 indels) were observed. Notably, in both the Illumina and ONT analyses, the proportion of errors was higher for indels compared to SNPs, in most samples, for both the analyses that included and excluded complex genomic regions.

**Table 3:** Comparison of WGS obtained by ONT and Illumina sequencing.

		ONT sequencing		Illumina sequencing	
		Positive control	dMDA with 5 pg input DNA	Positive control	dMDA with 5 pg input DNA
Number of reads		3 131 513	650 186	1 240 462 (× 2)	1 416 070 (× 2)
Mean genome-wide depth of coverage		452	147	65	61
Genome with ≥ 5 × depth of coverage (%)		99	99	98	98
Reads mapping to the H37Rv reference genome (%)		99	99	89	78
Lineage		L2.2.1.1	L2.2.1.1	L2.2.1.1	L2.2.1.1
Drug resistance profile		Pan-susceptible	Pan-susceptible	Pan-susceptible	Pan-susceptible
**Excluding complex regions**	**Total number of variants**	**1239**	**1238**	**1290**	**1293**
	Number of SNPs	1146	1153	1180	1180
	Number of indels	93	85	110	113
	**Number of false negative variants (%)**		**24 (1.94%)**		**1 (0.08%)**
	False negative SNPs		8 (0.65%)		1 (0.08%)
	False negative indels		16 (1.29%)		0 (0%)
	**Number of false positive variants (%)**		**23 (1.86%)**		**4 (0.31%)**
	False positive SNPs		15 (1.21%)		1 (0.08%)
	False negative indels		8 (0.65%)		3 (0.23%)
**Including complex regions**	**Total number of variants**	**1584**	**1596**	**1655**	**1657**
	Number of SNPs	1464	1482	1503	1503
	Number of indels	120	114	152	154
	**Number of false negative variants (%)**		**53 (3.32%)**		**31(1.87%)**
	False negative SNPs		32 (2.00%)		23 (1.39%)
	False negative indels		21 (1.32%)		8 (0.48%)
	**Number of false positive variants (%)**		**65 (4.07%)**		**33 (1.99%)**
	False positive SNPs		50 (3.13%)		23 (1.39%)
	False negative indels		15 (0.94%)		10 (0.60%)

*In this analysis, all variants identified by WGS analysis of the positive control sample were considered to be true variants, false positive variants were defined as variants observed in the dMDA-WGS sample but not in the positive control; false negative variants were defined as variants observed in the positive control sample but not detected in the dMDA-WGS sample.

## Discussion

In our study, we successfully applied dMDA to *Mtb* DNA using various input DNA amounts, ranging from 0.1 pg to 5 pg and achieved a 100,000 – 1,000,000 fold increase in the amount of DNA measured after performing dMDA. Variations in the depth of coverage were observed among the dMDA samples and the control and it was notable that the low-input dMDA samples exhibited greater fluctuation in coverage. However, achieving coverage uniformity with WGS in *Mtb* may be challenging due to the unique characteristics of the genome, and could be influenced by strain-specific genome characteristics such as repeat regions, large deletions, and duplications. The percentage of the genome covered with a minimum depth of 5 × emerges as a critical metric, essential for robust variant detection. This becomes particularly evident in scenarios such as that encountered in the 0.1 pg input dMDA sample, where a mere 44% of the genome achieved this depth of coverage. Consequently, in more than half of the genome, variants could not be reliably identified, drastically increasing the number of false negative variants. Interestingly, even with 0.1 pg of input DNA, we obtained a depth of genome coverage of 7 × , making it analysable when using the MAGMA pipeline and enabling accurate lineage identification, despite the large numbers of false positive and false negative variants observed. However, the results also indicated that dMDA could lead to non-specific amplification, with 52% and 64% mapped reads in the 0.1 pg and 0.5 pg input samples, respectively, suggesting that a large proportion of the generated sequencing reads do not map to the reference, potentially adding complexity to the analysis of results. This is supported by the increasing number of unclassified reads in dMDA samples, particularly noticeable with very low input *Mtb*-DNA, as evidenced by the taxonomic classification tool, Kraken 2's, inability to assign a taxonomic origin to increasing proportions of reads. There is thus a compelling need for the use of *Mtb* WGS analysis pipelines, like MAGMA^19,20^, that are capable of handling contaminants, whether originating from genuine contamination or artificially introduced MDA artefacts. While MDA has not yet been evaluated for NGS of *Mtb*, MDA has been used successfully to increase the amount of genomic DNA from clinical smear-negative sputum specimens before processing the DNA with standard IS*6110*-specific PCR methods^30^.

Our findings complement the promising results of the application of dMDA for WGA for bacterial species, suggesting that dMDA can offer significant improvements in amplification efficiency and specificity compared to conventional methods, making it a valuable tool for bacterial whole genome amplification^31,32^. For example, in the study by Rhee et al. (2016), dMDA achieved a 65-fold increase in coverage and more than a 20-fold increase in specificity when compared to conventional tube MDA for a sample containing 0.1 pg/μl *of E. coli* DNA^31^. Hosokawa et al*.* (2017) demonstrated the effectiveness of dMDA in preventing contamination and amplification bias in single-cell whole genome amplification of mixed *E. coli* and *B. subtilis* strains encapsulated in droplets^32^.

An important observation of our study was the impact of the quantity of DNA used for dMDA on the accuracy of variant calling. MAGMA analysis of the Illumina data revealed that dMDA resulted in an unacceptably high number of false positive and false negative variants when low amounts (0.1 pg or 0.5 pg) of input DNA were amplified. These erroneous variants likely stem from limitations inherent to the MDA method, as underscored by Rhee et al*.*^31^. False positive variants predominantly arise due to early polymerase errors that are amplified because of low template copy numbers, whereas false negative variations primarily result from reduced depth and breadth of coverage with unsequenced regions preventing variants from being identified. Careful consideration is thus needed when interpreting the results, as non-specific amplification and false variants can occur at low DNA input levels. It is also worth noting that this approach may not be suitable for the detection of minor (unfixed) variants, as dMDA introduces false positive minor variants, even at higher input DNA concentrations.

Following the currently established guidelines for the interpretation of variant differences ^33^, an input DNA threshold of ≥ 5 pg emerges as the minimum requirement to ensure reliable and sufficient amplification of *Mtb* DNA for accurate variant identification for drug resistance assessment and phylogenetic investigations. In parallel, our investigation into ONT sequencing with the positive control and 5 pg input dMDA sample revealed noteworthy results. We observed a higher proportion of false positive and false negative variants with ONT sequencing compared to Illumina sequencing using the same input DNA amount. It's important to note that the potential errors introduced by MDA and the lower per base sequence accuracy of ONT sequencing compared to Illumina, introduce a layer of complexity into the variant calling process. These findings emphasize the intricacies inherent in variant identification when dealing with data generated from MDA techniques, shedding light on the potential challenges associated with precise variant determination under such conditions.

Despite the promising results of dMDA combined with Illumina sequencing, some limitations need to be acknowledged. Firstly, the control sample was sequenced on an Illumina MiniSeq, while the dMDA samples were sequenced on an Illumina iSeq, which may explain differences in average base quality between the control and dMDA samples. Secondly, we used diluted purified DNA extracted from a sub-cultured *Mtb* clinical isolate using a time-consuming and laborious method for our experiments representing a controlled research laboratory setting, which may not fully capture the complexities associated with clinical samples such as mixed DNA populations and varied DNA extraction efficiencies. To further validate the method's applicability in real-world clinical scenarios, future investigations should include experiments with clinical samples, incorporating the complexities of paucibacillary sputum samples and extrapulmonary samples, and applying rapid and efficient DNA extraction techniques that have the potential to be automated. Thirdly, our ability to compare Illumina to ONT sequencing was limited to the 5 pg sample. This limitation arises from ONT's requirement for substantial DNA quantities, resulting in the entire sample being allocated for Illumina library preparation. Therefore, the comparative assessment between these sequencing platforms was constrained by the available sample size. In addition, the variant identification accuracy of each sequencing approach was inferred in relation to the approaches’ positive control, the advantage of this approach is that the accuracies specifically reflect the effect of the dMDA on variant identification. Future experiments could repeat this comparison for a reference sample where the variants are precisely established, as such the accuracy would reflect not only the effect of the dMDA but also of the other factors such as sequencing technology and pipeline on the total accuracy.

To further improve the use of dMDA for clinical samples, coupling dMDA with ONT's adaptive sampling approach could be an interesting avenue to explore as adaptive sampling allows for real-time enrichment of specified target DNA during sequencing^34^. Alternatively, another approach to consider involves combining dMDA with Selective Whole Genome Amplification (SWGA) primers. SWGA employs primers designed to specifically target the *Mtb* genome, as illustrated by Clarke et al*.* in 2017^35^. This strategy opens the possibility of applying dMDA in clinical samples containing DNA other than *Mtb* to overcome the overamplification of non-*Mtb* DNA.

In conclusion, dMDA shows promise as a robust and efficient method for WGS of bacterial DNA, including *Mtb* DNA. Starting from minute amounts of ≥ 5 pg of pure *Mtb* input DNA extracted from a cultured clinical isolate, the dMDA method can sufficiently and accurately amplify *Mtb*-DNA for WGS-based drug resistance inference, phylogeny and possibly the assessment of transmission. Further research and application of dMDA in clinical samples are essential to validate its performance and reliability as a diagnostic method.

### Supplementary Information

Supplementary Information.

## Data Availability

Raw Illumina and ONT sequencing data of the dMDA-WGS and the positive control sample were deposited to the European Nucleotide Archive under accession number: PRJEB55560.
