# Supplementary material for: Droplet based whole genome amplification for sequencing minute amounts of purified Mycobacterium tuberculosis DNA
Source: Sci Rep. 2024 Apr 30;14:9931. doi: 10.1038/s41598-024-60545-1 (PMC11061190; doi:10.1038/s41598-024-60545-1)
Supplement: Supplementary file 1 — Supplementary Information. [file 41598_2024_60545_MOESM1_ESM.pdf]

SUPPLEMENTARY MATERIAL: APPENDIX 1

Droplet Based Whole Genome Amplification for Sequencing Minute Amounts of Purified *Mycobacterium tuberculosis* DNA

Table S1. False positive and false negative variants in *pe/ppe* genes for Illumina dMDA samples

False positive and False negative variants in *pe/ppe* genes				
**dMDA *Mtb* input DNA:**	**0.1 pg**	**0.5 pg**	**1 pg**	**5 pg**
Total number of FN variants (incl. complex regions)	758	113	54	31
of which in *pe/ppe* genes (%)	150 (20%)	59 (52%)	36 (67%)	23 (74%)
Total number of FP variants (incl. complex regions)	209	146	45	33
of which in *pe/ppe* genes (%)	27 (13%)	24 (16%)	28 (62%)	18 (55%)

FN=false negative; FP=false positive

Table S2. Illumina WGS read classification with Kraken 2

Sample	% unclassified reads	% Total reads: Bacteria	% MTBC reads
Control	14.35	85.65	85.12
5 pg	17.08	82.61	81.74
1 pg	16.63	83.08	81.17
0.5 pg	22.26	76.91	74.01
0.1 pg	24.85	73.59	63.33

Allele frequency analysis:

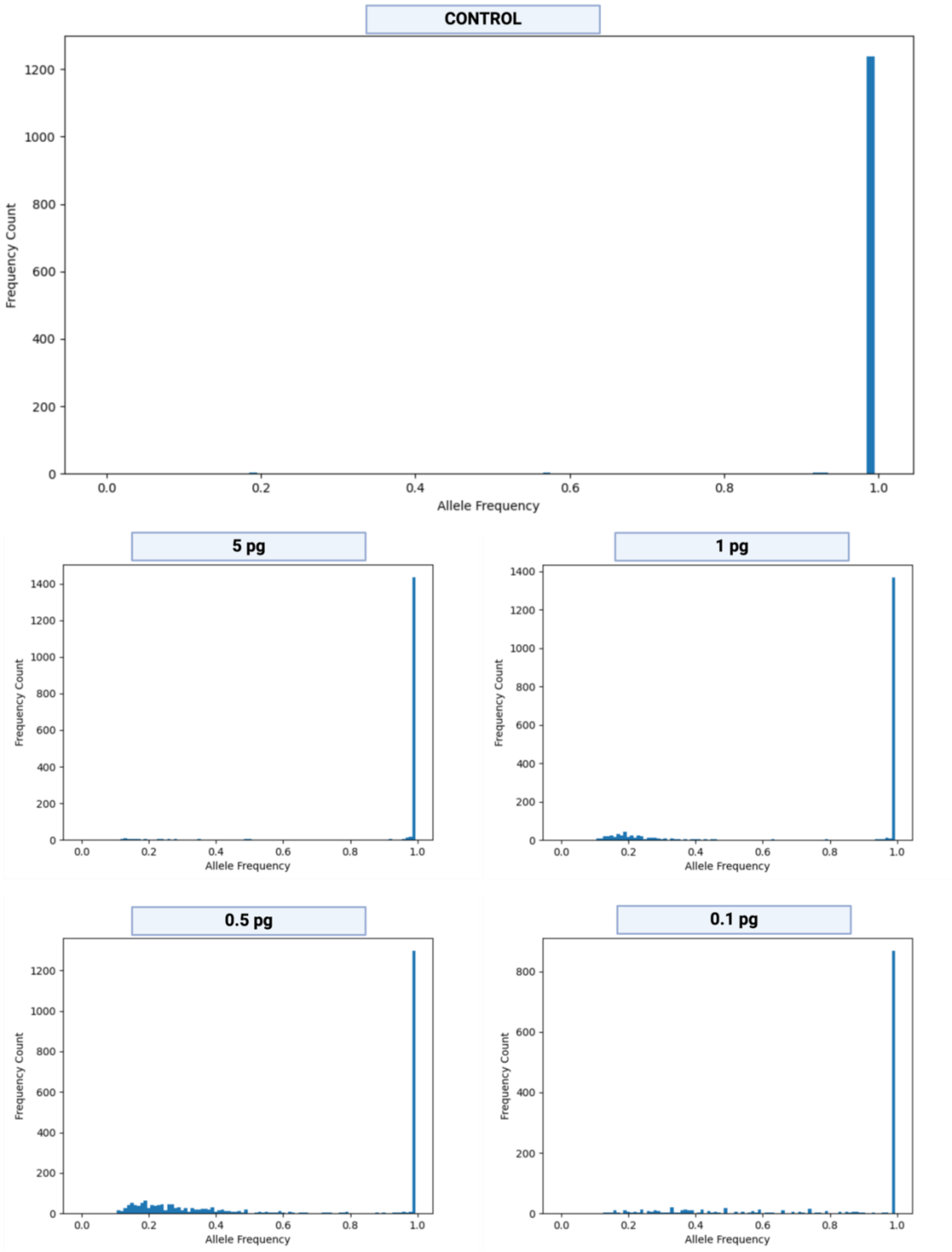

Figure S1. Allele Frequency Distribution of Variants in Control and dMDA Samples. Histograms displaying the distribution of allele frequencies of unfiltered variants in the control sample and each of the dMDA samples. The x-axis represents the allele frequency, while the y-axis indicates the count of variants falling within each allele frequency bin.

Genome-wide depth of coverage plots:

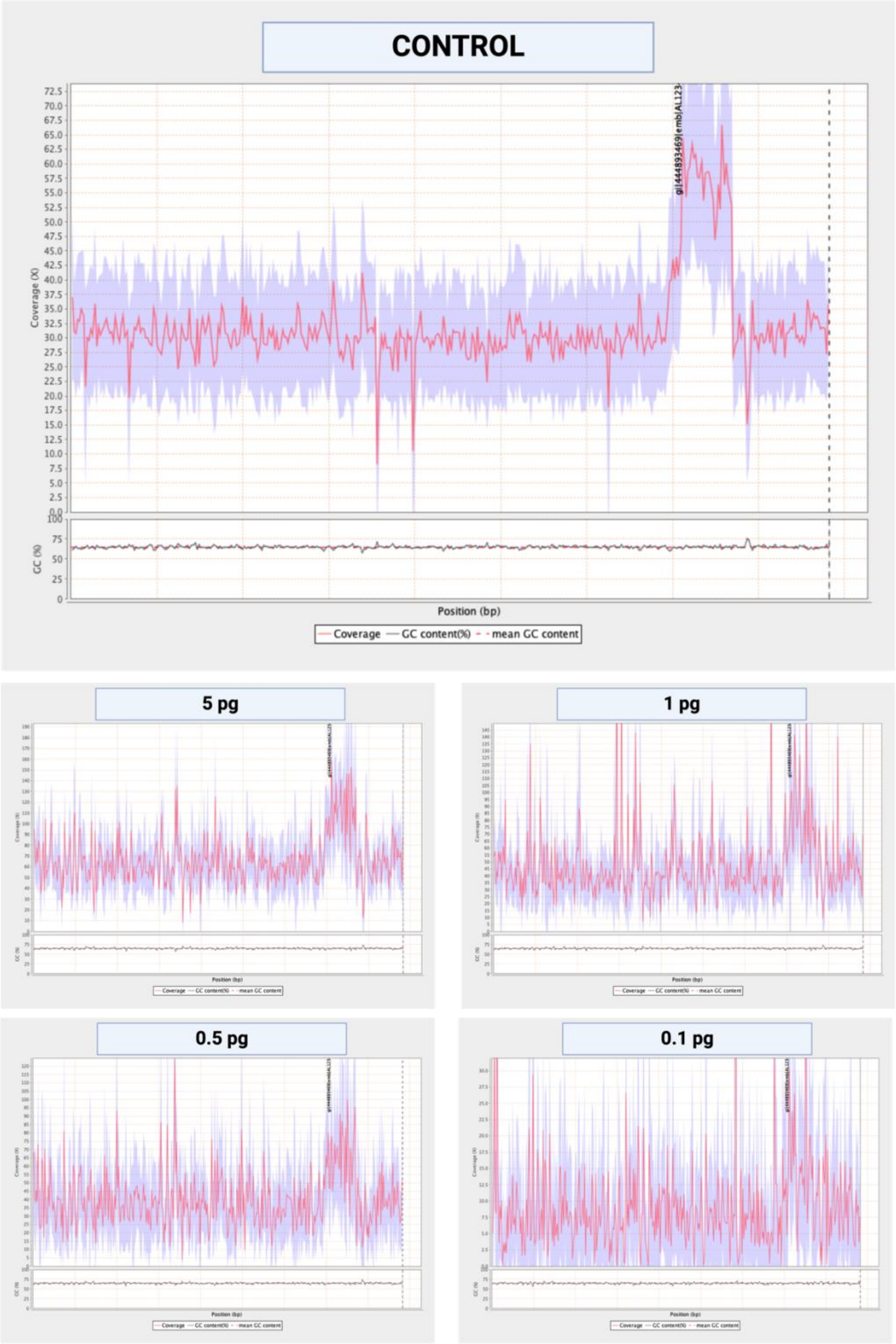

Figure S2. Genome-wide depth of coverage plots. The genome-wide depth of coverage was plotted for the control sample, and 5 pg, 1 pg, 0.5 pg, and 0.1 pg input *Mtb* DNA samples, respectively. The y-axis represents the depth of coverage, while the x-axis indicates the genome position. Additionally, the GC-composition is depicted below each depth of coverage plot.
